# Integrating natural product research laboratory with artificial intelligence: Advancements and breakthroughs in traditional medicine

**DOI:** 10.37796/2211-8039.1475

**Published:** 2024-12-01

**Authors:** Jai-Sing Yang, Shih-Chang Tsai, Yuan-Man Hsu, Da-Tian Bau, Chia-Wen Tsai, Wen-Shin Chang, Sheng-Chu Kuo, Chien-Chih Yu, Yu-Jen Chiu, Fuu-Jen Tsai

**Affiliations:** a Department of Medical Research, China Medical University Hospital, China Medical University, Taichung, Taiwan; b Department of Biological Science and Technology, China Medical University, Taichung, Taiwan; c Department of Animal Science and Biotechnology, College of Agriculture and Health, Tunghai University, Taichung, Taiwan; d Graduate Institute of Biomedical Sciences, China Medical University, Taichung, Taiwan; e Terry Fox Cancer Research Laboratory, Department of Medical Research, China Medical University Hospital, Taichung, Taiwan; f Drug Development Center, China Medical University, Taichung, Taiwan; g School of Pharmacy, College of Pharmacy, China Medical University, Taichung, Taiwan; h Division of Plastic and Reconstructive Surgery, Department of Surgery, Taipei Veterans General Hospital, Taipei, Taiwan; i Department of Surgery, School of Medicine, National Yang Ming Chiao Tung University, Taipei, Taiwan; j Million-Person Precision Medicine Initiative, Department of Medical Research, China Medical University Hospital, Taichung, Taiwan; k School of Chinese Medicine, College of Chinese Medicine, China Medical University, Taichung, Taiwan; l China Medical University Children's Hospital, Taichung, Taiwan; m Department of Medical Genetics, China Medical University Hospital, Taichung, Taiwan

**Keywords:** Natural Products Research Laboratories (NPRL), Artificial Intelligence (AI), Natural products, Drug research and development (R&D), Drug discovery

## Abstract

The Natural Product Research Laboratory (NPRL) of China Medical University Hospital (CMUH) was established in collaboration with CMUH and Professor Kuo-Hsiung Lee from the University of North Carolina at Chapel Hill. The laboratory collection features over 6000 natural products worldwide, including pure compounds and semi-synthetic derivatives. This is the most comprehensive and fully operational natural product database in Taiwan. This review article explores the history and development of the NPRL of CMUH. We then provide an overview of the recent applications and impact of artificial intelligence (AI) in new drug discovery. Finally, we examine advanced powerful AI-tools and related software to explain how these resources can be utilized in research on large-scale drug data libraries. This article presents a drug research and development (R&D) platform that combines AI with the NPRL. We believe that this approach will reduce resource wastage and enhance the research capabilities of Taiwan's academic and industrial sectors in biotechnology and pharmaceuticals.

## 1. Introduction

Living organisms such as plants, invertebrates, and microorganisms produce chemical molecules known as natural products. These compounds exhibit a range of biological and pharmacological activities, including anti-cancer, antioxidant, anti-aging, and anti-inflammatory properties, making them valuable for the research and development (R&D) of new drugs [1–4]. Identifying bioactive compounds typically involves several steps: obtaining natural products from biological sources, testing their efficacy as medicines, isolating bioactive substances, determining their structures, identifying their molecular targets, and utilizing bioinformatics for further analysis [5]. However, these traditional research models are often time-consuming and require significant financial investment.

**Fig. 1:**
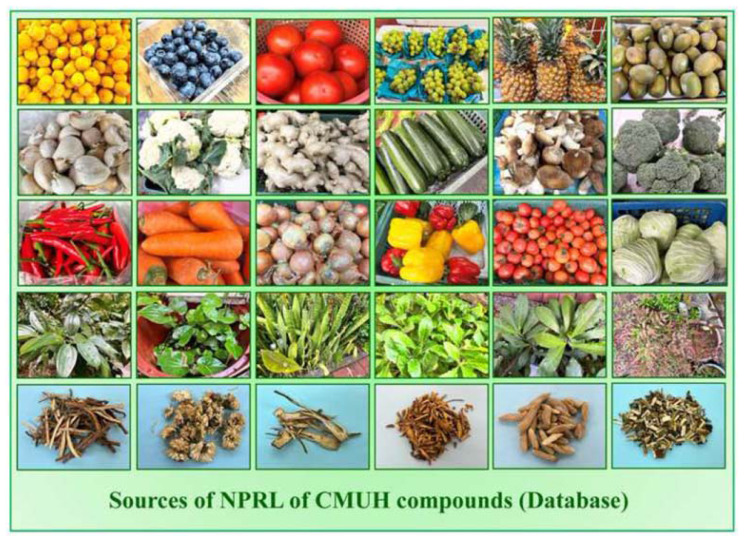
Sources of NPRL of CMUH compounds database. The NPRL of CMUH utilizes a variety of natural compounds. This collection includes plant-based materials, such as fruits, vegetables, herbs, roots, and plants used in traditional medicine. The samples ranged from everyday food items such as tomatoes, carrots, garlic, and ginger to rare herbs and medicinal plants being investigated for their potential therapeutic properties. NPRL utilizes these varied resources as fundamental materials for extracting and developing natural compounds, facilitating drug discovery efforts and supporting studies in pharmacological research.

To keep pace with evolving trends in new drug development, it is essential to establish a large-scale, integrated compound database R&D platform in Taiwan, especially given the rapid advancements in artificial intelligence (AI) and biotechnology [6–8]. The Natural Product Research Laboratory (NPRL) database aims to create a comprehensive resource of compound big data, employ high-speed AI computational tools to develop potential lead compounds, and facilitate clinical applications in translational medicine [9–12]. China Medical University Hospital (CMUH) and Professor Kuo-Hsiung Lee are funding the NPRL-CMUH initiative, which focuses on sourcing compounds from fruits, vegetables, microorganisms, traditional Chinese medicine (TCM), and Chinese herbal materials (Fig. 1). Detailed information on NPRL-CMUH is provided in the following sections.

This review gathers relevant literature on the use of artificial intelligence (AI) tools and techniques in drug discovery applied throughout all phases of drug development. These methods aim to expedite the research process while minimizing the risks and costs of clinical trials.

## 2. Summary of history and process for constructing NPRL of CMUH

### 2.1. Development and establishment for the NPRL of CMUH: A comprehensive approach to constructing a natural product compound database

The development of the NPRL compound database was initiated with a planning stage in 2017 and finalized in 2019, spanning three years [13–15]. A crucial aspect of establishing NPRL is the comprehensive documentation and cataloging of all the compounds. A thorough action plan was formulated before the commencement of the project. Academics from CMUH collaborated with Professor Lee's research group to examine the various elements of these compounds, including their storage requirements, physical locations, individual scientific documentation, and related publication lists. Our team gathered general project details and identified potential challenges that might arise during the process. For example, we discovered that certain compounds and experimental data were stored in separate containers, necessitating meticulous verification. We also addressed issues related to the storage and preservation of specific compounds, particularly those that require preparation before shipment back to Taiwan. Furthermore, we developed a specialized AI software application to inventory all existing and future compounds within the NPRL.

Establishing this software is crucial before successfully entering the compound data.

### 2.2. Systematic organization and documentation of NPRL compounds

**Fig. 2:**
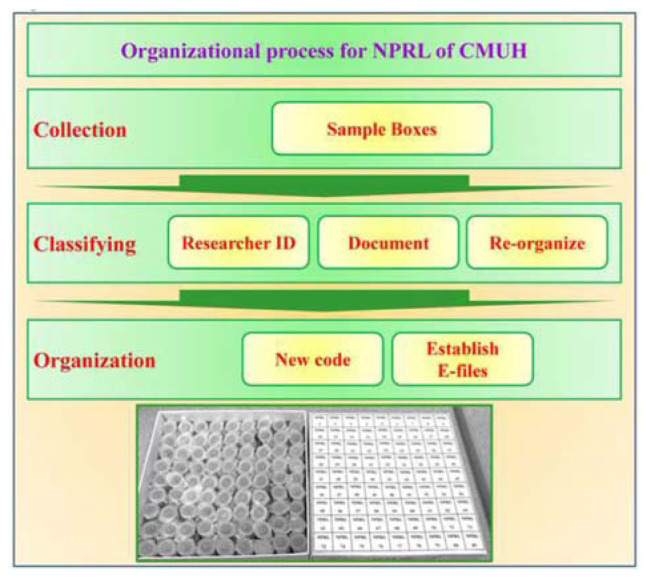
Organizational process for the NPRL of CMUH. The structured approach for establishing NPRL of CMUH outlines the steps for acquiring, categorizing, and arranging compound specimens. Acquisition: the initial phase involved obtaining sample containers containing diverse compounds. Categorization: specimens are sorted based on crucial information, such as the investigator's identity, pertinent paperwork, and reorganization specifications. This approach ensures precise and effortless retrieval. Arrangement: each specimen is given a unique identity, and digital records are generated to support computerized data management and streamline future access.

Our initial approach involved establishing a primary method for systematically organizing and documenting NPRL compounds. The process illustrated in Fig. 2 began with collecting all sample containers. Once gathered, the containers were sorted according to their designated names. Subsequently, we meticulously inventoried the contents of each container and correlated them with existing experimental data, various documents, and digital records. The final step involved assigning unique identification codes to individual samples. The NPRL of CMUH database requires the inclusion of specific details for each entry. These include a unique NPRL Code Number, the Original Code Number, CID Number, PubChem/CAS Number, and Common Name. Additionally, the structure, molecular weight, and quantity must be determined. The Sample State (e.g., solid or liquid) and Sample Location (e.g., freezer or storage box) are also required. Finally, any related publications and other data pertinent to entry should be included.

### 2.3. Development of NPRL: Taiwan's largest natural products database and AI-driven drug R&D

**Fig. 3:**
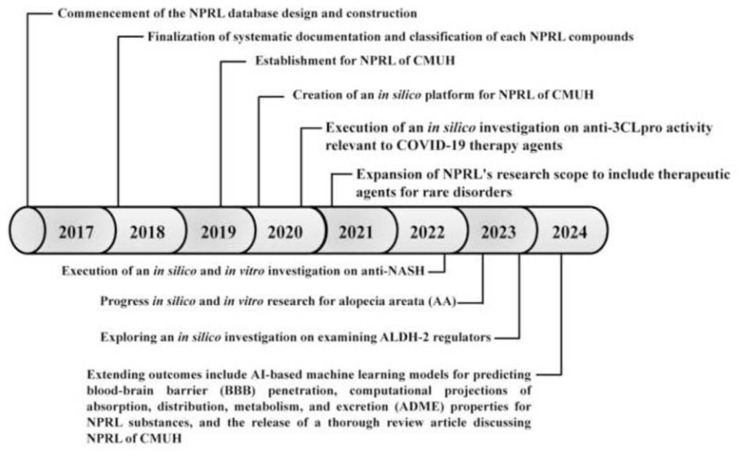
Chronology of NPRL evolution and utilization. The timeline of significant events, developmental phases, and scientific applications associated with the NPRL of CMUH from 2017 to 2024.

The NPRL of CMUH repository contains 6782 natural products, including both pure and semi-synthetic derivatives, making it Taiwan's most extensive and comprehensive collection of natural products. Through structural refinement, 37,682 compounds with diverse configurations were obtained. The development of NPRL was finalized in 2019, after which work began on creating *in silico* and *in vitro* platforms for various medical conditions, thereby enabling the expansion of this substantial database. Fig. 3 illustrates the construction process, research initiatives, and current research directions for NPRL from 2017 to 2024.

- 2017: Commencement of the NPRL database design and construction.
- 2018: Finalization of systematic documentation and classification of NPRL compounds.
- 2019: Establishment for NPRL of CMUH.
- 2020: Creation of an *in silico* platform for NPRL of CMUH.
- 2020: Execution of an *in silico* investigation on anti-3CLpro activity relevant to COVID-19 therapy agents.
- 2021: Expansion of NPRL's research scope to include therapeutic agents for rare disorders.
- 2022: Execution of an *in silico* and *in vitro* investigation on anti-NASH.
- 2023: Progress *in silico* and *in vitro* research for alopecia areata (AA) therapeutic agents.
- 2023: Execution of an *in silico* investigation on examining ALDH-2 regulators with NPRL compounds.
- 2024: Additional progress and research outcomes include AI-based machine learning (ML) models for predicting blood–brain barrier (BBB) penetration, computational projections of ADME (absorption, distribution, metabolism, and excretion) properties for NPRL-CMUH substances, and the release of a thorough review article discussing NPRL at CMUH.

**Fig. 4:**
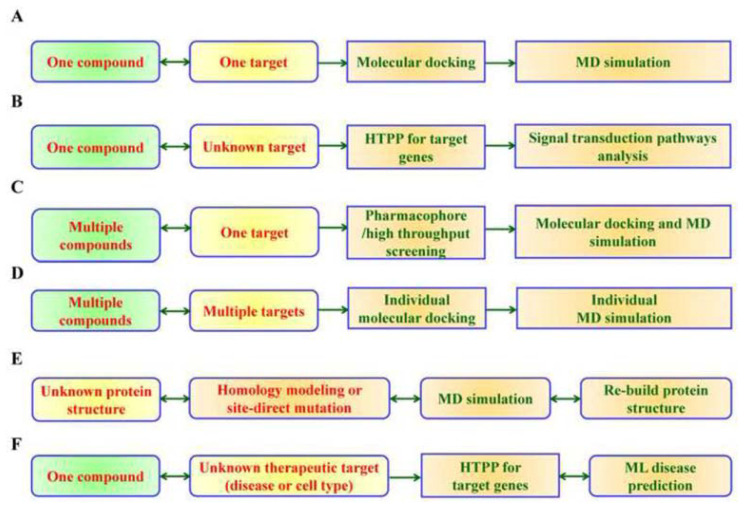
Pipeline of In Silico R&D platforms for various disease models at NPRL. Each component (A–F) showed a distinct computational approach for analyzing compounds and targets. (A) Single-compound single-target model: Molecular docking was used to test a single compound against a specific target, and MD simulation was used to improve the interaction analysis. (B) Single compound unknown target model: HTPP is used to identify possible target genes for compounds with unknown targets. A signal transduction pathway analysis is added to clarify the compound's mode of action. (C) Multiple compound-single target model: pharmacophore modeling or high-throughput screening tests of a group of compounds against a single target. Molecular docking and MD simulations are then used for a more in-depth analysis. (D) The multiple compound-multiple target model tests different compounds against different targets. Molecular docking and MD simulations improve the connection between each compound and its target. (E) Homology Modeling and site-directed mutation prediction model: Homology modeling or site-directed mutagenesis was used for proteins with unknown structures or mutations. This is followed by MD simulations and protein structure reconstruction. (F) Therapeutic Target Identification Model: If the compound's therapeutic target (disease or cell type) is unknown, HTPP is used to guess the target genes. ML techniques are used to treat diseases.

Table 1 presents a compilation of websites and databases containing extensive compound libraries relevant to pharmaceutical R&D [13,16–30]. Fig. 4 presents a comprehension design of AI-driven *in silico* drug R&D platform that CMUH created to address various medical conditions. The system is composed of different parts: (A) a compound connected to a model for predicting a target, (B) a compound connected to a model for predicting an unidentified target, (C) a library of compounds linked to a model for predicting a target, (D) multiple compounds combined with a model for predicting multiple targets, (E) a model for homology modeling or site-directed mutation prediction, and (F) models for finding combined therapeutic targets. Fig. 4 shows the AI-driven *in silico* drug R&D platform of CMUH, which was developed to address various diseases.

## 3. AI applications in drug R&D

### 3.1. Advancing drug development: Utilizing AI for efficient pharmaceutical R&D

Recently, AI has made substantial progress across various social domains with notable advancements in the pharmaceutical sector [31,32]. AI encompasses diverse, sophisticated tools, including reasoning capabilities, knowledge representation systems, solution search algorithms, and networking technologies [33]. The pharmaceutical industry has witnessed the extensive digitization of experimental and clinical data over the past few decades, enabling the analysis and processing of big data [33,34]. By implementing AI modules, the sector can improve the automation of large-scale data management and proactively tackle potential future issues, enabling the earlier identification of solutions [35]. Traditionally, drug development has relied on identifying and creating numerous small molecule compounds. Various compound libraries have been established over the last decade. Conventional approaches to drug development are often expensive and time-intensive, hampering the process's efficiency. Nevertheless, AI can address these challenges. This study examined AI techniques for drug R&D [33–36].

**Fig. 5:**
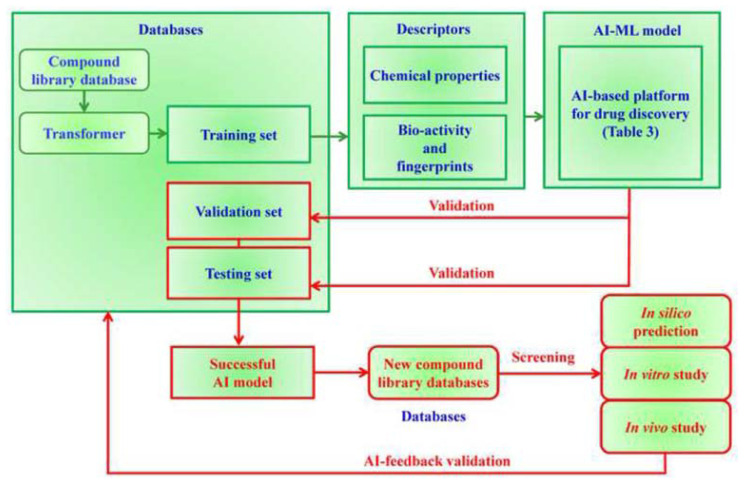
AI-powered drug discovery leveraging compound library databases. The process consists of three main phases: 1. Database management and data preparation; 2. Model development and verification; and 3. Model application and screening. The drug discovery workflow can be optimized using AI-based modeling and validation techniques, enabling more effective screening and prediction of potential therapeutic agents.

Fig. 5 provides a detailed illustration of the workflow of the AI model. The process began with data extraction from the compound library database, followed by using a transformer to separate the data into training, validation, and testing sets. The training set comprised chemical characteristics, biological activity, and molecular fingerprints subjected to computational analysis and ML [13,37]. Validation and testing sets were used to verify the results. Once the AI model has been successfully validated, it can be used to analyze other compound library database. Commonly used ML methods for developing classification models in AI include linear discriminant analysis (LDA), k-nearest neighbors (kNN), kNN regression (kNNR), artificial neural networks (−), probabilistic neural networks (PNN), support vector machines (SVM), support vector regression (SVR), C4.5 decision trees (C4.5DT), recursive partitioning (RP) classifiers, random forests (RF), naïve Bayes classifiers, multiple linear regression (MLR), partial least squares regression (PLSR), and logistic PLS, among others. Several AI-driven platforms for discovering novel pharmaceuticals are listed in Table 2 [38–47].

### 3.2. AI-driven approaches for disease classification and novel drug development

**Fig. 6:**
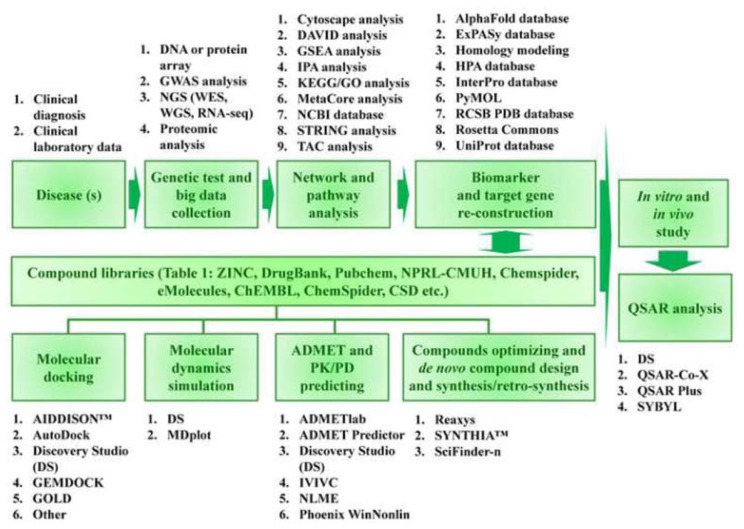
Workflow of AI-enhanced drug discovery and development processes. This diagram showcases a holistic AI-based drug discovery and development strategy, from disease identification to experimental testing utilizing various computational and empirical methodologies. The process is divided into eight crucial phases: 1. Disease identification and data collection; 2. Network and pathway examination; 3. Biomarker and target gene reconstruction; 4. Compound library screening; 5. Molecular docking and dynamics simulation; 6. ADMET and PK/PD property prediction; 7. Compound optimization and novel design; and 8. QSAR analysis and experimental verification. This approach effectively integrates AI and computational techniques with traditional research methods to accelerate drug discovery, improve candidate selection, and enhance the efficacy of experimental validation.

Standard analytical software processes large compound libraries with AI-driven models. Fig. 6 and Table 3 present an overview of the workflow for AI-enhanced drug discovery and development, along with a list of commonly used software programs. Disease classification can be achieved by compiling the clinical diagnoses and laboratory findings. Following Institutional Review Board (IRB) approval for specimen collection, researchers can investigate the relationship between diseases and genes [48–50]. Various techniques have been employed, including DNA or protein arrays, Genome-Wide Association Studies (GWAS), and next-generation sequencing (NGS) methods, such as whole-exome sequencing (WES), whole-genome sequencing (WGS), RNA sequencing, and proteomic analysis [51–55]. Examination of genetic big data, encompassing network and pathway analyses, enables the identification of disease biomarkers and target genes. These analyses utilize multiple approaches, such as Cytoscape analysis, DAVID analysis, Gene Set Enrichment Analysis (GSEA), Ingenuity Pathway Analysis (IPA), KEGG/GO analysis, MetaCore analysis, NCBI database utilization, STRING analysis, and TCA analysis. Various protein structure repositories, including the Alpha-Fold database, Binding MOAD, ExPASy, HPA, InterPro, RCSB PDB, Rosetta Commons, and Uni-Prot, allow access to known crystal structures [49,50,56–72]. AI-based homology modeling techniques can be employed to predict the three-dimensional configurations [14,73].

Various software tools have been employed for molecular docking between molecules and proteins, including AutoDock, AIDDISON™, Discovery Studio (DS), GEMDOCK, GOLD, and PotentialNet. Following docking, programs such as DS and MDplot can be utilized to perform molecular dynamics simulations. Upon confirming the binding affinity of a compound to a protein, additional characteristics, such as pharmacokinetics/pharmacodynamics (PK/PD) and blood–brain barrier (BBB) permeability, can be estimated using platforms such as ADMETlab, ADMET Predictor, DS, IVIVC, NLME, and Phoenix WinNonlin. The creation of innovative molecular structures is crucial in novel drug development. Software such as Reaxys, SYNTHIA ™, and SciFinder-n can be used for compound optimization, *de novo* compound design, and synthesis/retrosynthesis [14,74–89].

**Fig. 7:**
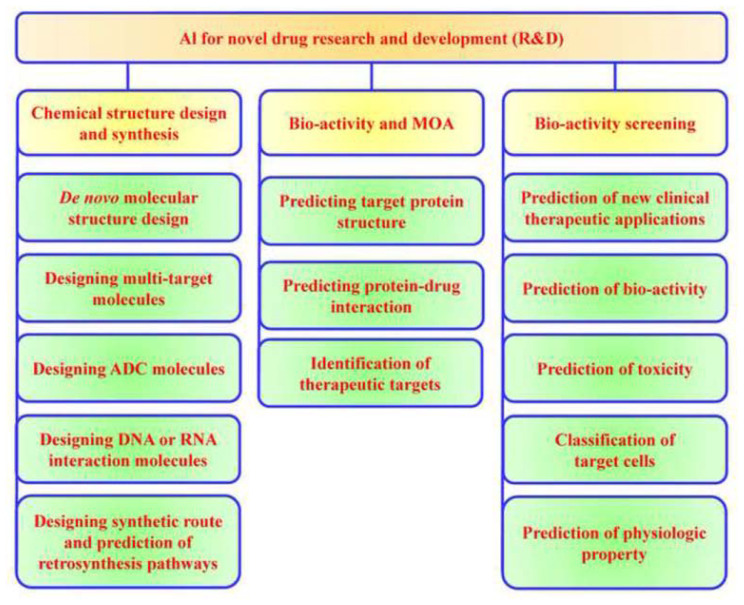
AI applications in drug development: Improving the efficiency of chemical design, bioactivity assessment, and predictive screening. The field of novel medication research and development employs three main categories of AI applications, each with a specific role: (1) Chemical structure design and synthesis, (2) Bioactivity and MOA, and (3) Bioactivity screening and prediction. AI plays a crucial role in various stages of drug development, including molecular design, therapeutic outcome forecasting, and safety profile evaluation. These AI-driven applications shorten the research and development process, enhancing prospects for achieving successful outcomes.

Fig. 7 outlines the roles and benefits of AI in novel drug R&D. AI-driven tools offer diverse capabilities that significantly enhance the new drug discovery process. These tools facilitate the creation of novel molecular structures, the development of multi-target compounds, and the generation of antibody-drug conjugates (ADCs) [90,91]. They also support the design of nucleic acid (DNA or RNA)-interacting compounds, streamline chemical synthesis, and enable the reverse engineering of chemical structures, thus transforming various facets of chemical and molecular design [92,93]. In the field of bioactivity and mechanism of action (MOA) research, AI assists in predicting target protein structures, modeling protein–drug interactions, and identifying potential therapeutic targets [94,95]. Furthermore, AI technologies are instrumental in uncovering new clinical applications through bioactivity screening, assessing bioactivity and toxicity, classifying target cells, and predicting physiological properties [6,8,32].

## 4. Conclusion

**Fig. 8:**
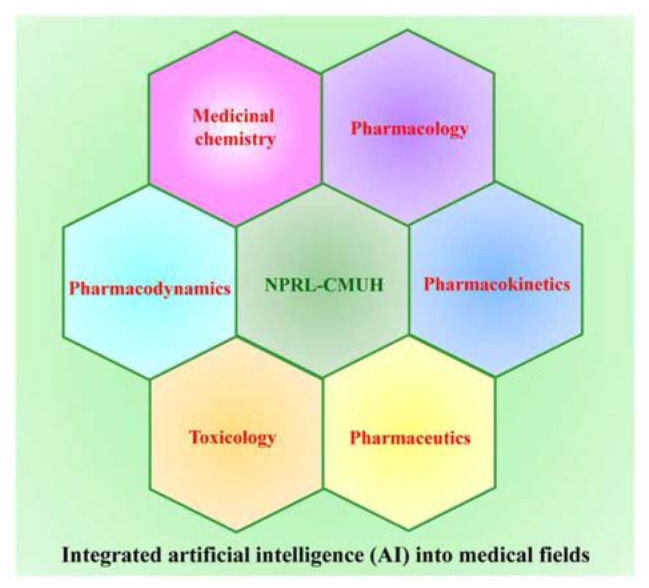
Impact of AI integration on NPRL of CMUH and related pharmaceutical fields. Integrating AI within the NPRL framework significantly impacts various pharmaceutical disciplines, including medicinal chemistry, pharmacology, pharmacokinetics, pharmacodynamics, toxicology, and pharmaceutics. Incorporating AI fosters a more efficient, data-driven approach to drug discovery and development, ultimately enhancing patient care and medical treatments.

Having the right tools is essential for achieving excellence in any task. Table 4 outlines the benefits and prospects for creating an NPRL of CMUH database. Combining NPRL compounds with AI technology can significantly improve the creation of new small-molecule structures, uncover novel therapeutic targets, and reveal new pharmacological uses of natural product lead compounds. This synergy offers crucial insights for treating various human diseases and developing new drugs, ultimately enhancing patient care and quality of life. Integrating AI into pharmacies within NPRL is anticipated to spur progress and innovations in fields such as medicinal chemistry, pharmacology, pharmacodynamics, pharmacokinetics, toxicology, and pharmaceutics (Fig. 8).

## Figures and Tables

**Table 1:** Websites and databases of large compound libraries for drug research and development (R&D).

Name	Type	Website URL	References
ClinicalTrials.gov	Clinical trials database	https://www.clinicaltrials.gov/	[16]
Chemical entities of biological Interest (ChEBI)	Small chemical molecule database	https://www.ebi.ac.uk/chebi/	[17]
ChEMBL database	Database of bioactive molecules	https://www.ebi.ac.uk/chembl/	[18]
ChemSpider The cambridge structural	Chemical molecule database	https://www.chemspider.com/	[19]
Database (CSD)	Chemical molecule database	https://www.ccdc.cam.ac.uk/solutions/software/csd/	[20]
DailyMed database	FDA-regulated products	https://dailymed.nlm.nih.gov/dailymed/	[21]
DrugBank	Drug database	https://go.drugbank.com/	[22]
Drugs@FDA	FDA approved drugs database	https://www.accessdata.fda.gov/scripts/cder/daf/index.cfm	[25]
FDA online label repository	FDA drug label database	https://labels.fda.gov/	[23]
FooDB IUPHAR/BPS guide to	Nature products database	https://foodb.ca/	[24]
Pharmacology	Pharmacology database	https://www.guidetopharmacology.org/	[26]
PubChem	Chemical molecule database	https://pubchem.ncbi.nlm.nih.gov/	[27]
PKIDB	Kinase inhibitor database	https://www.icoa.fr/pkidb/	[28]
TargetMol	Natural products database	https://www.targetmol.com/search?keyword=home/	[29]
TCMBank	Traditional Chinese medicines	https://tcmbank.cn/	[30]
ZINC	Available compounds	zinc.docking.org/	[13]

**Table 2:** AI-based tools and platform for drug research and development (R&D).

Platform name	Website URL	References
ADMET-AI	https://flask.palletsprojects.com/en/2.3.x/	[39]
BBBP	https://paperswithcode.com/dataset/bbbp-scaffold	[40]
BrainMaker	http://www.calsci.com/	[38]
BSVM	http://www.csie.ntu.edu.tw/~cjlin/bsvm/	[38]
DeepChem	https://github.com/deepchem/deepchem	[41]
Dense K nearest neighbor	http://www.autonlab.org/autonweb/10522.html	[38]
DeltaVina	https://github.com/chengwang88/deltavina	[42,43]
e1071 R package	http://cran.r-project.org/web/packages/e1071/index.html	[38]
Fast random forest	https://code.google.com/p/fast-random-forest/	[38]
Fann	http://leenissen.dk/fann/	[38]
GPU-FS-kNN	http://sourceforge.net/projects/gpufsknn/	[38]
GA/KNN	http://www.niehs.nih.gov/research/resources/software/biostatistics/gaknn/	[38]
Hit dexter	http://hitdexter2.zbh.uni-hamburg.de	[44,45]
KNN	http://www.fit.vutbr.cz/~bartik/Arcbc/kNN.htm	[38]
K nearest neighbor demo	http://www.cs.cmu.edu/~zhuxj/courseproject/knndemo/KNN.html	[38]
LIBSVM	http://www.csie.ntu.edu.tw/~cjlin/libsvm/	[38]
LS-SVMlab	http://www.esat.kuleuven.be/sista/lssvmlab/	[38]
MolProphet	https://www.molprophet.com/login	[46]
mySVM	http://www-ai.cs.uni-dortmund.de/SOFTWARE/MYSVM/index.html	[38]
M-SVM	http://www.loria.fr/~guermeur/	[38]
NuClass	http://www.uta.edu/faculty/manry/new_software.html	[38]
NVIDIA BioNeMo™	https://docs.nvidia.com/clara/index.html	[47]
NVIDIA MegaMolBART	https://github.com/NVIDIA/MegaMolBART	[13]
OC1	http://www.cbcb.umd.edu/~salzberg/announce-oc1.html	[38]
PC4.5	http://www.cs.nyu.edu/~binli/pc4.5/	[38]
Random forests	http://www.stat.berkeley.edu/~breiman/RandomForests/	[38]
Random forest R package	http://cran.r-project.org/web/packages/randomForest/index.html	[38]
Simple decision tree	https://sites.google.com/site/simpledecisiontree/	[38]
Sciengyrpf	http://sourceforge.net/projects/sciengyrpf/	[38]
Sharky neural network	http://sharktime.com/us_SharkyNeuralNetwork.html	[38]
SMILES	http://users.dsic.upv.es/~flip/smiles/	[38]
SVM light	http://svmlight.joachims.org/	[38]
XGBoost	https://xgboost.readthedocs.io/en/stable/	[13]
YaDT	http://www.di.unipi.it/~ruggieri/software.html	[38]

**Table 3:** Common analytical methods and software for analyzing big data in compound libraries with AI models.

Type	Software	Website URL	References
Network and pathway analysis	Cytoscape (3.10.3)	https://cytoscape.org/	[56]
	DAVID (v2024q2)	https://david.ncifcrf.gov/home.jsp	[57]
	GSEA (MSigDB 2024.1)	https://www.gsea-msigdb.org/gsea/index.jsp	[58]
	IPA (v107193442)	https://digitalinsights.qiagen.com/products-overview/discoveryinsights-portfolio/analysis-and-visualization/qiagen-ipa/	[49,50]
	KEGG analysis	https://www.genome.jp/kegg/	[59]
	Gene ontology	https://geneontology.org/	[60]
	MetaCore	https://clarivate.com/lp/metacore-integrated-pathway-analysis-for-multi-omics-data/	[61]
	NCBI	https://www.ncbi.nlm.nih.gov/	[62]
	STRING (v12.1)	https://string-db.org/	[63]
	TAC (v4.0.1)	https://www.thermofisher.com/tw/zt/home/life-science/microarray-analysis/microarray-analysis-instruments-software-services/microarray-analysis-software/affymetrix-transcriptome-analysis-console-software.html	[64]

Biomarker and target gene re-construction	AlphaFold (v3)	https://alphafold.ebi.ac.uk/	[65]
	ExPASy	https://www.expasy.org/	[66]
	HPA	https://www.proteinatlas.org/	[67]
	InterPro (v102.0)	https://www.ebi.ac.uk/interpro/	[68]
	PyMOL (v3.0)	https://pymol.org/	[69]
	RCSB PDB	https://www.rcsb.org/	[70]
	Rosetta commons	https://rosettacommons.org/	[71]
	UniProt	https://www.uniprot.org/	[72]

Molecular docking	AIDDISON™	https://www.merckgroup.com/en/research/science-space/envisioning-tomorrow/future-of-scientific-work/aiddison.html	[74]
	AutoDock (v4.2.6 and GPU)	https://autodock.scripps.edu/	[75]
	Discovery studio (2024)	https://www.3ds.com/products/biovia/discovery-studio	[14]
	GEMDOCK	http://gemdock.life.nctu.edu.tw/	[76]
	GOLD	https://www.ccdc.cam.ac.uk/solutions/software/gold/	[77]
	NovaDock	https://www.dnastar.com/software/nova-protein-modeling/novaDock/	[78]
	Other		[79]

Molecular simulation	Dynamics discovery studio (2024)	https://www.3ds.com/products/biovia/discovery-studio	[14]
	MDplot	https://github.com/MDplot/MDplot	[80]

ADMET and PK/PD Predicting	ADMETlab (v3.0) ADMET	https://admetlab3.scbdd.com/	[81]
	predictor	https://www.simulations-plus.com/software/admetpredictor/	[82]
	Discovery studio (2024)	https://www.3ds.com/products/biovia/discovery-studio	[14]
	Phoenix™ IVIVC Toolkit	https://www.certara.com/software/phoenix-ivivc-toolkit/	[83]
	NLME (Phoenix™	https://www.certara.com/software/phoenix-nlme/	[84]
	Platform v8.5.1)		
	Phoenix WinNonlin	https://www.certara.com/software/phoenix-winnonlin/	[85]

Compounds optimizing reaxys and de novo compound SYNTHIA™		https://www.reaxys.com/#/login	[86]
		https://www.merckgroup.com/en/research/science-space/envisioningTomorrow/future-of-scientific-work/synthia.html	[87]
Design	SciFinder-n	https://www.cas.org/solutions/cas-scifinder-discovery-platform/cas-scifinder	[86]

QSAR analysis	Discovery studio (2024)	https://www.3ds.com/products/biovia/discovery-studio	[14]
	QSAR-Co-X	https://github.com/ncordeirfcup/QSAR-Co-X	[88]
	QSAR plus	https://www.3ds.com/products/biovia/discovery-studio/qsar-admet-predictive-toxicology	[96]
	SYBYL	https://sybyl-x.software.informer.com/2.1/	[89]

**Table 4:** Advantages and opportunities in establishing the NPRL of CMUH database.

Advantages	The number of samples includes up to 6782 distinct types
	Cultivate research talents in nature products, phytochemicals, Chinese herbal and traditional Chinese medicine (TCM)
	Enrich the diversity of research resources in nature products, phytochemicals, Chinese herbal and TCM
	Receive support from leading research centers both domestically and internationally
	Advanced research outcomes in the fields of TCM and natural products
	Facilitate the integration of Chinese and Western medicine
	Accelerate the pace of new drug discovery
	Establish a foundation for international collaboration
Opportunities	Establish AI models for TCM diseases and treatments
	Utilize innovative technologies and methods
	Enhance the applications of translational medicine
	Advance drug discovery and patent technology transfer
	Increase research outcomes and publications in TCM
	Create job opportunities for TCM professionals
	Expand avenues for international collaboration
	Improve patient health and well-being

## List of abbreviations

AA: Alopecia areata
ADC: Antibody-drug conjugates
AI: Artificial intelligence
AlphaFold: Alphafold protein structure database
CMUH: China Medical University Hospital
DAVID: Database for Annotation, Visualization and Integrated Discovery
DS: Discovery studio
E-files: Electronic files
ExPASy: Expert protein analysis system
GO: Gene ontology
GOLD: Genetic Optimisation for Ligand Docking
GSEA: Gene set enrichment analysis
GWAS: Genome-Wide Association Study
HPA: The human protein atlas
HTPP: High-throughput protein production
IPA: Ingenuity pathway analysis
IRB: Institutional Review Board
IVIVC: *In vivo in vitro* correlation
MD: Molecular dynamics
Metacore: Metacore enrichment analysis
ML: Machine learning
MOAD: Mother of all databases
NASH: Non-alcoholic steatohepatitis
NCBI: National center for biotechnology information
NGS: Next generation sequencing
NPRL: Natural Product Research Laboratory
NLME: WinNonMix
PD: Pharmacodynamics
PK: Pharmacokinetics
PPB: Plasma protein binding
QSAR: Quantitative structure-activity relationship
RCSB_PDB: RCSB protein data bank
R&D: Research and development
SMILES: Simplified molecular input line entry system
STRING: Search tool for the retrieval of interacting genes
TAC: Transcriptome analysis console
UniProt: Universal Protein
WES: Whole exome sequencing
WGS: Whole genome sequencing
RNA-seq: RNA sequencing
